# Renal K^+^ retention in physiological circumstances: focus on adaptation of the distal nephron and cross-talk with Na^+^ transport systems

**DOI:** 10.3389/fphys.2023.1264296

**Published:** 2023-08-31

**Authors:** Samia Lasaad, Gilles Crambert

**Affiliations:** ^1^ Centre de Recherche des Cordeliers, Institut National de la Santé et de la Recherche Médicale, Sorbonne Université, Université Paris Cité, Laboratoire de Physiologie Rénale et Tubulopathies, Paris, France; ^2^ CNRS EMR 8228—Unité Métabolisme et Physiologie Rénale, Paris, France

**Keywords:** potassium balance, ATP12A, blood pressure, sodium balance, extracellular compartment

## Abstract

Consumption of salt (NaCl) and potassium (K^+^) has been completely modified, switching from a rich-K^+^/low-NaCl diet in the hunter–gatherer population to the opposite in the modern, westernized population. The ability to conserve K^+^ is crucial to maintain the plasma K^+^ concentration in a physiological range when dietary K^+^ intake is decreased. Moreover, a chronic reduction in the K^+^ intake is correlated with an increased blood pressure, an effect worsened by a high-Na^+^ diet. The renal adaptation to a low-K^+^ diet in order to maintain the plasma K^+^ level in the normal range is complex and interconnected with the mechanisms of the Na^+^ balance. In this short review, we will recapitulate the general mechanisms allowing the plasma K^+^ value to remain in the normal range, when there is a necessity to retain K^+^ (response to low-K^+^ diet and adaptation to gestation), by focusing on the processes occurring in the most distal part of the nephron. We will particularly outline the mechanisms of K^+^ reabsorption and discuss the consequences of its absence on the Na^+^ transport systems and the regulation of the extracellular compartment volume and blood pressure.

## General background

Potassium (K^+^) is the most abundant cation present in our body and is essential to several physiological functions, such as cell volume regulation, maintenance of the membrane resting potential, and cardiac and neuromuscular activities. The total body K^+^ content is kept constant at around 50–55 mmol K^+^/kg body weight. About 98% of the K^+^ present in the body is intracellular (stored in the muscle, liver, and erythrocytes), and it is the main cation of our cells with an intracellular concentration of 120 mmol/L. The plasma concentration of K^+^ must be maintained within very narrow physiological limits between 3.5 mmol/L and 5 mmol/L, regardless of the daily intake. An excess of K^+^ in the extracellular fluids (hyperkalemia, plasma K^+^ value > 5 mmol/L) decreases the plasma membrane potential. Conversely, K^+^ deficiency (hypokalemia, plasma K^+^ value <3.5 mmol/L) induces hyperpolarization of the membrane. Both these conditions are pathological and reflect an imbalance in potassium homeostasis, leading to muscular and/or neurological disorders that can lead to death in severe cases.

This partition between extra- and intracellular compartments is due to the electrochemical gradient of K^+^ across the cell membrane generated by Na^+^, K^+^-ATPase. Na^+^, K^+^-ATPase is a ubiquitous heterodimeric transmembrane protein that hydrolyzes ATP to pump three Na^+^ ions out of the cells and two K^+^ ions into the cells, which generates and maintains the electrochemical gradients of Na^+^ and K^+^. The pump is composed of two subunits, namely, the catalytic subunit called the ⍺-subunit, a ten-transmembrane helix domain, and the β-subunit, a smaller glycosylated single-transmembrane domain. There are multiple isoforms of both the ⍺ (⍺1 to ⍺2) and β subunits (β1 to β3), which exhibit a different tissue distribution. These isoforms can combine to create different Na^+^, K^+^-ATPase isozymes that possess different transport and pharmacological characteristics (Crambert et al., 2000).

**FIGURE 1:**
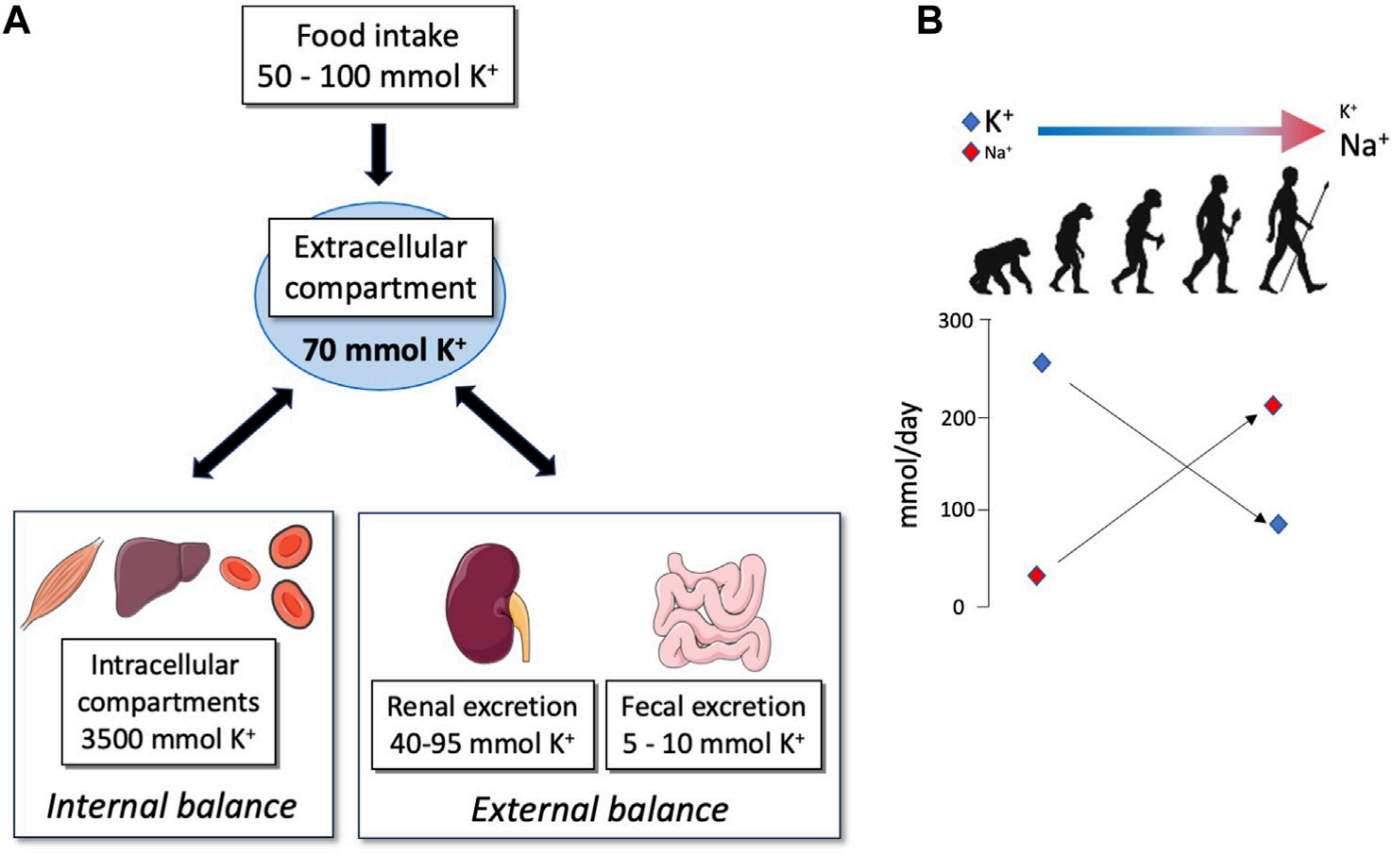
**(A)** Schematic representation of K^+^ repartition in different compartments of the organism. The priority of the organism is to maintain the extracellular K^+^ concentration into a narrow range despite the variations in K^+^ intake; this value corresponds to 70 mmol of K^+^ for a human weighing about 70 kg. For this purpose, K^+^ may be pumped or released from internal stores (internal balance) or may be reabsorbed or secreted in the feces and urine (external balance) in order to excrete exactly the amount of K^+^ ingested. **(B)** Evolution of Na^+^ and K^+^ intakes adapted from Meneton et al. (2004).

Potassium balance (Figure 1A) is regulated by many different mechanisms involving internal [storage or release into or from cellular reservoir like muscles; for review, see McDonough et al. (2002)] and external (renal and intestinal excretion) regulatory systems. In these integrated and orchestrated systems, the kidney (specifically the most distal parts of the nephron) plays a central role (Doucet and Crambert, 2015). Under normal physiological situations, as a result of the equilibrium between processes of reabsorption and secretion, the amount of K^+^ excreted by the kidney roughly corresponds to the amount ingested (Giebisch et al., 2003).

The modern Western diet is characterized by the consumption of processed foods with a high intake of protein, high-fat dairy products, and high-sugar drinks, which correlate with the development of metabolic disorders (diabetes and obesity). This diet also has profound but often underappreciated impacts on electrolyte balance. Indeed, a typical Western diet produces approximately 50 mmol of acid/day whereas our ancestors were considered to be the net base producers (Sebastian et al., 2002). In parallel, the consumption of salt (NaCl) and K^+^ has been completely changed, shifting from a rich-K^+^/low-NaCl diet in hunter–gatherer populations to the opposite (low-K^+^/rich NaCl diet) in the modern, westernized population (Figure 1B) (Meneton et al., 2004). In Western countries, the daily potassium intake is much lower than that recommended by the international boards and health organizations (4.7 g or 120 mmol), with values that are between 40 and 70 mmol/day (Vega-Vega et al., 2018; Blanchard et al., 2020). Interestingly, the daily potassium intake has been established as a determinant of blood pressure (BP). Global association study analyses clearly showed that a decrease in K^+^ intake is correlated with an increase in BP and morbidity (Mente et al., 2014; O'Donnell et al., 2014), suggesting that variations in K^+^ intake may interfere with the Na^+^ balance. Therefore, understanding how regulations of K^+^ balance, in the context of dietary restriction, interfere with the Na^+^ balance is crucial.

## Mechanisms of K^+^ secretion in the distal parts of the nephron

**FIGURE 2:**
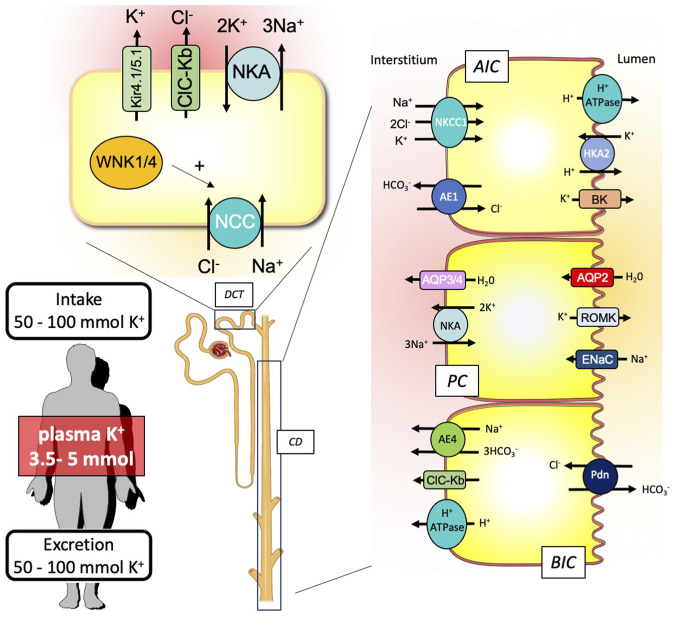
Schematic representation of the renal key players involved in the external K^+^ balance. To maintain equilibrium between the K^+^ input and output, the kidney is equipped with nephron segments, the distal convoluted tubule (DCT), and the collecting duct (CD) that contribute to K^+^ secretion or reabsorption. CD is composed of different cell types expressing specific ion and water transporters: the A-type intercalated cells (AICs), the principal cells (PCs), and the B-type intercalated cells (BICs).

Although the transepithelial transport of K^+^ occurs all along the nephron, the mechanisms accounting for establishing the K^+^ balance take place primarily at the distal convoluted tubule (DCT), the connecting tubule (CNT), and the collecting duct (CD) (Figure 2).

Regarding K^+^ secretion, the crucial event is the creation of a favorable transepithelial potential through the stimulation of the epithelial Na^+^ channel (ENaC). This allows the luminal exit of K^+^ from the principal cells through K^+^ channels (Kir1.1 also known as ROMK). Another K^+^ channel with a higher conductance (BK channel) than ROMK is also important for K^+^ secretion (Liu et al., 2011). Although they are mainly expressed in intercalated cells and require a signal to be activated and opened (increase of luminal flow that triggers the increase in intracellular Ca^2+^), the passage of K^+^ through these channels is also dependent on a negative transepithelial voltage and, therefore, on the ENaC activity (Cornelius et al., 2015). This stimulation of ENaC activity is mainly dependent on aldosterone, which is produced in response to an increase in the extracellular K^+^ concentration. However, this is not sufficient to induce the electrogenic Na^+^ transport at the apical side of the principal cells. The Na^+^ delivery at the collecting duct is also a crucial parameter. Under normal conditions, the Na^+^ concentration in the collecting duct is rather low since it has been reabsorbed upstream, in the DCT, by the Na/Cl exchanger (NCC). To promote K^+^ secretion, it is, therefore, important that the Na^+^ delivery is increased in the lumen of the collecting duct to provide enough Na^+^ to be reabsorbed by ENaC. Thus, Sorensen et al. showed that the inhibition of NCC actually preceded ENaC activation by aldosterone or proteolytic cleavages (Sorensen et al., 2013). Recently, the group of Ellison has proposed an elegant model, suggesting that the variations in the plasma K^+^ concentration are sensed by DCT cells, leading to proportional variations in the intracellular Cl^−^ concentration (Terker et al., 2015). The with-no-lysine kinases (WNKs) that control the activity of NCC are sensitive to intracellular Cl^−^ and, therefore, to modifications of extracellular K^+^ concentrations. Thus, when the plasma K^+^ concentration increases, the intracellular Cl^−^ concentration increases as well, which turns NCC activity off. This switch between the electroneutral and electrogenic Na^+^ transport systems (NCC in the DCT vs ENaC in the CCD) also allows the body to globally maintain the Na^+^ balance at equilibrium, when K^+^ secretion is required, since the amount of Na^+^ that is not reabsorbed in the DCT is reabsorbed in the CCD.

## Mechanisms of K^+^ reabsorption in the distal nephron

The presence of two types of H, K-ATPase in the kidney has puzzled experts in the field of renal ion transport systems for many years. These transporters belong to the large family of P-type ATPases, forming with the Na, K-ATPase isoforms the subgroup of the so-called X,K-ATPases. The H,K-ATPase type 1, also known as the “gastric” H,K-ATPase, is present in the kidney, where it could participate in the renal adaptation to metabolic acidosis. As for type 2, it has been referred to as the “non-gastric” or “colonic” H,K-ATPase and exhibits intriguing transport properties, and pharmacological and physiological features when compared to type 1 [for review, see Crambert (2014)]. In the kidney, its expression is extremely low under normal conditions but is strongly stimulated after K^+^ depletion (Ahn et al., 1996; Marsy et al., 1996; Kraut et al., 1997). Since HKA2 is stimulated under a low-K^+^ diet, many investigators proposed that this transporter should play a role in allowing the kidney to efficiently retain K^+^ under K^+^ depletion (see the next section). In 1998, Meneton et al. (1998) showed that HKA2-null mice under basal or K^+^-depleted conditions exhibit a colonic loss of K^+^ compared to their wild-type littermates. However, the renal handling of K^+^ was similar in WT and HKA2-null mice, leading to the conclusion that the presence of renal HKA2 was not required for maintaining the K^+^ balance even during K^+^ restriction. However, we described physiological situations in which the stimulation of renal HKA2 is required (Salhi et al., 2012; Salhi et al., 2013a). Moreover, El Moghrabi et al. (2010) showed that tissue kallikrein participates in the secretion of K^+^ after a K^+^ load by inhibiting HKA2, therefore indicating that this pump is active in the kidney.

## Renal mechanisms of K^+^ conservation under dietary K^+^ restriction

When the organism faces a lack of K^+^, the pathways described previously are regulated in order to inhibit its secretion and to enhance its reabsorption.

### Mechanisms promoting the inhibition of renal K^+^ secretion

The inhibition of K^+^ secretion in the collecting duct has been attributed to two different but complementary mechanisms: 1)a direct inhibition of ROMK and 2)the inhibition of the lumen-negative transepithelial potential generated by the activity of ENaC. Regarding the first point, the group of Wang has established, with a series of publications (for review see (Wang and Giebisch, 2008)), that the increase in intracellular reactive oxygen species (ROS) during the K^+^ depletion triggers the inhibition of ROMK, following a cascade of event involving kinases such as c-Src, ERK, p38, and WNK1.

On the other hand, K^+^ restriction strongly and rapidly decreases the plasma aldosterone concentration, leading to a decreased activity of ENaC. Palmer and co-workers were the first to establish that, in this condition, Na^+^ balance is maintained because of the activation of NCC, upstreaming the collecting duct (Frindt and Palmer, 2010; Frindt et al., 2011). If the decreased activity of ENaC is easily explained by the well-established decrease in aldosterone, the mechanism inducing the parallel activation of NCC in response to K^+^ restriction was less obvious to establish. The sensing of a decrease in extracellular K^+^ by the DCT cells through the modification of activities of K^+^ and Cl^−^ channels (Kir4.1/Kir5.1 and ClC-Kb, respectively) is a key pathway to activate WNK4 and induced phosphorylation of NCC (Terker et al., 2015). Kristensen et al. showed that the increased NCC activity is indeed directly dependent on the low extracellular K^+^ and does not require any hormonal stimulus (Kristensen et al., 2022). The increased reabsorption of Na^+^ by the DCT that decreases the Na^+^ delivery at the collecting duct and thus decrease the ENaC activity and K^+^ secretion is also related to a morphological modification of the DCT. By using a tissue clearing approach, it was shown that DCT cells proliferate, increasing the size of this segment, which may be related to an increased capacity of Na^+^ reabsorption (Saritas et al., 2018).

### Mechanisms promoting the stimulation of renal K^+^ reabsorption

Up to now, two processes, possibly complimentary, have been described to stimulate HKA2 expression during a dietary K^+^ restriction in the distal part of the nephron (Ahn et al., 1996; Marsy et al., 1996; Kraut et al., 1997). The first process involved the ROS produced during K^+^ depletion in the collecting duct (as mentioned previously; for review, see Wang (2006)) that activate the transcription factor Nrf2, which is shown to enhance the expression of HKA2 (Lee et al., 2012). In 2011, we described a novel pathway that participates at the stimulation of HKA2 after chronic K^+^ depletion (Elabida et al., 2011). We, indeed, observed that chronic K^+^ depletion modifies the adrenal steroidogenesis and leads to the production and release of progesterone in both male and female animals. Using different approaches, we have, then, demonstrated that progesterone induced the renal expression of HKA2. The inhibition of the progesterone action blocks the stimulation of HKA2 and results in a renal loss of K^+^. The elevation of plasma progesterone in response to a low-K^+^ diet was confirmed later (Keppner et al., 2019) in mice under a low-K^+^ diet. We investigated this process in humans mildly depleted in K^+^ after a controlled dietary restriction or in severely hypokalemic patients (Gitelman syndrome). The mild reduction in K^+^ intakes did not impact the plasma concentration of progesterone or that of its metabolites. However, in hypokalemic patients suffering from Gitelman syndrome, steroidogenesis was also strongly modified and its activation by ACTH revealed that these patients were more prone to produce progesterone than healthy volunteers (Blanchard et al., 2020).

The direct activation of K^+^ reabsorption also requires modification of CD composition. Thus, the number of type A intercalated cells (AICs) expressing HKA2 (Constantinescu et al., 1997; Kraut et al., 2001) is increased in response to dietary K^+^ restriction in mice (Cheval et al., 2004; Duong Van Huyen et al., 2008). The mechanisms behind this increased number of AIC were described as involving a proliferation (Cheval et al., 2004) process and/or interconversion from principal cells (Park et al., 2012). GDF15, a TGFβ-related growth factor, was identified by transcriptomic analysis as one of the most upregulated genes in the CD from mice after strict dietary K^+^ restriction depleted in K^+^ (Cheval et al., 2004) and in response to an acid load (Cheval et al., 2006). This growth factor was, then, shown to be produced by the principal cells of the CD in response to acidosis and to trigger AIC proliferation in this condition (Duong Van Huyen et al., 2008). Recently (Lasaad et al. manuscript in press), we showed that GDF15 expression was increased in all renal tubular segments and parts of the intestine and colon of mice in response to the low-K^+^ diet. This broad overexpression led to a systemic elevation of its plasma and urine concentrations. In GDF15 knock-out mice, the renal adaptation to a low-K^+^ diet is delayed compared to WT, which is related to the absence of AIC proliferation. The action of GDF15 on AIC is sensitive to mubritinib, a specific inhibitor of an ErbB2 receptor. Interestingly, the overexpression of HKA2, expected in response to a low-K^+^ diet, was completely blunt in GDF15 knock-out mice. Using HKA2 knock-out mice, we finally observed that this pump is important for cell proliferation since these mice under a low-K^+^ diet did not increase their AIC number as WT did. There is, therefore, a pathway that links principal cells, sensing K^+^ restriction and producing GDF15, and AIC proliferation that requires the presence of HKA2.

## Plasma K^+^ levels versus the volume of the extracellular compartment, we have to choose!

The processes implemented to inhibit the secretion of K^+^ and its reabsorption should work together and be complementary. We can suppose that the absence of one could be compensated by the other. Thus, the absence of HKA2, which prevents the reabsorption of K^+^ and the proliferation of AIC, could be compensated by a greater inhibition of the secretion system (greater activation of NCC, for example,). However, under the low-K^+^ diet or during gestation when the K^+^ reabsorption process is inhibited as in the HKA2 knock-out mice, this compensatory scenario is not observed (Walter et al., 2016; Walter et al., 2020).

### Adaptation to a low-K^+^ diet when K^+^ reabsorption is impeded

Indeed, HKA2 knock-out mice, in response to a rather short dietary K^+^ restriction (4 days), exhibited a clear hypokalemia (conversely to WT mice that remained normokalemic) but did not exhibit stimulation of NCC as WT did. Therefore, despite the observed hypokalemia, DCT of HKA2 knock-out mice does not reabsorb more Na^+^. This leads to a slight and transient increase in renal Na^+^ excretion and to the development of hypovolemia. This result was not expected since, as mentioned previously, hypokalemia should stimulate NCC expression according to Elisson’s model (Terker et al., 2015). In addition to extracellular K^+^, vasopressin has also been involved in the stimulation of NCC (Castaneda-Bueno et al., 2014). We confirmed this action of vasopressin by showing that the treatment of WT mice under a low-K^+^ diet with a V2 receptor antagonist inhibited the activation of NCC (Walter et al., 2016). Investigation of the vasopressin system in more detail in HKA2 knock-out mice during K^+^ restriction showed a resistance to vasopressin, leading to a decreased expression of AQP2 after 4 days of K^+^ restriction, which is related to an increase in PGE2 production (known to antagonize vasopressin effects (Chabardes et al., 1988). So, the less efficient action of vasopressin and the absence of stimulation of NCC in HKA2 knock-out mice lead to a loss of fluid that decreases the volume of the extracellular compartment and blood pressure (Walter et al., 2016).

### Adaptation to gestation when K^+^ reabsorption is impeded

During normal gestation, the volume of the extracellular compartment is increased due to the stimulation of Na^+^ reabsorption in CCD (West et al., 2010). Interestingly, in parallel, NCC activity is downregulated (West et al., 2015). This situation with high ENaC activity and low NCC activity resembles the adaptation to a K^+^ load that would permit to excrete a large amount of K^+^. However, during gestation, K^+^ is also retained (Atherton et al., 1982; Lindheimer et al., 1987) despite the ENaC-mediated Na^+^ reabsorption, which is counterintuitive. In 2013, we established that the K^+^ retention observed during gestation was mediated by the activation of HKA2, which was confirmed later (Salhi et al., 2013b; West et al., 2018). The absence of HKA2 was indeed detrimental to the gestation and interferes with the K^+^ balance of the gravid HKA2 knock-out female animals, but surprisingly, their plasma K^+^ value did not decrease as expected (Salhi et al., 2013b). It turns out, that in the absence of HKA2, the Na^+^ reabsorption through ENaC activation is blunted, resulting in a lower extension of the extracellular volume, which leads to a decrease in the blood pressure of the gravid HKA2 knock-out mice (Walter et al., 2020). The lack of ENaC activation in gravid HKA2 knock-out mice may be related to their lower production of aldosterone compared to gravid WT mice. Indeed, since these knock-out mice need to retain K^+^ through a mechanism that cannot involve HKA2, their only solution is to reduce K^+^ secretion by decreasing the aldosterone level. However, they also need to increase their extracellular volume by retaining Na^+^ and fluid. This leads to a paradoxical situation where the increase in aldosterone is blunted, limiting the extension of the extracellular compartment. Here again, the reduction in the volume of the extracellular compartment seems to be favored to maintain the plasma K^+^ concentration in the normal range when K^+^ retention processes are impeded.

Thus, in these two situations where HKA2-mediated K^+^ reabsorption is required, the absence of this pathway is not compensated by a stronger inhibition of the secretion but by a reduction in the extracellular compartment volume. When the organism faces the risk of non-manageable hypokalemia, a physiological response could consist in reducing the plasma volume (hypovolemia) to the detriment of blood pressure to concentrate K^+^ in the extracellular compartment.
